# Knowledge, Utilization, and Perceptions of Artificial Intelligence in Medical Education Among Preclinical Osteopathic Medical Students and Faculty: A Cross-Sectional Study

**DOI:** 10.7759/cureus.109675

**Published:** 2026-05-26

**Authors:** Raju Panta, Laura Francois, Hassan Cordash, Jake Orent

**Affiliations:** 1 Physiology and Pathology, Burrell College of Osteopathic Medicine, Melbourne, USA; 2 College of Medicine, Burrell College of Osteopathic Medicine, Melbourne, USA; 3 College of Medicine, Burrell College of Osteopathic Medicine, Las Cruces, USA

**Keywords:** ai knowledge and use, artificial intelligence, attitudes toward use of ai, future perspectives of use of ai, medical education, osteopathic medical students, perception of use of ai

## Abstract

Introduction

Artificial intelligence (AI), including generative large language models such as ChatGPT and Microsoft Copilot, is increasingly incorporated into medical education. However, empirical data describing AI knowledge, utilization, and perceptions within osteopathic medical education remain limited. This study aimed to assess AI literacy, usage patterns, perceived benefits and concerns, and future readiness among preclinical osteopathic medical students and faculty.

Methods

A cross-sectional, anonymous, web-based survey was administered to first- and second-year osteopathic medical students and preclinical faculty at Burrell College of Osteopathic Medicine across two campuses. Survey items assessed demographics, AI knowledge and training, educational and scholarly AI use, perceptions, attitudes toward verification and policy, and future perspectives. Descriptive statistics summarized responses, and chi-square tests compared faculty and student groups.

Results

Ninety‑four responses were received (26.0% response rate), with 58 complete responses (20 faculty and 38 students) included in analyses. Formal AI training was uncommon overall, but faculty were significantly more likely than students to have received any formal AI training (35.0% vs. 2.6%; p = 0.003). Students demonstrated significantly higher use of AI for studying or lecture preparation (76.3% vs. 45.0%; p = 0.036), generating exam questions (63.2% vs. 20.0%; p = 0.002), reviewing exam questions (71.1% vs. 10.0%; p < 0.001), completing written assignments (36.8% vs. 0%; p = 0.005), and writing patient notes (92.1% vs. 5.0%; p < 0.001). Students were also more likely to use AI‑driven digital anatomy platforms (81.6% vs. 35.0%; p = 0.001). Students more frequently identified treatment planning as an area that would benefit from AI integration (31.6% vs. 5.0%; p = 0.048).

Conclusions

This study provides preliminary institutional insights into AI knowledge, utilization, and perceptions among preclinical osteopathic medical students and faculty at a single osteopathic medical school. Respondents demonstrated moderate baseline AI knowledge and strong interest in future integration, with students showing substantially higher use of AI tools than faculty. These early patterns underscore a need for structured AI literacy education, targeted faculty development, and the establishment of institutional governance to guide responsible and pedagogically sound AI adoption within this educational context.

## Introduction

Artificial intelligence (AI) has rapidly emerged as a transformative force in modern healthcare, influencing diagnostic accuracy, clinical decision-making, workflow optimization, and health professions education [1-3]. Contemporary AI systems incorporate advances in machine learning (algorithms that identify patterns from data using statistical models), deep learning (a specialized subset of machine learning that uses multilayer neural networks to automatically learn hierarchical representations), and natural language processing to analyze large and diverse datasets, including electronic health records, biomedical literature, medical imaging, and genomic data, with performance approaching or exceeding human expertise in selected applications [3,4]. Rather than replacing clinicians, AI is increasingly conceptualized as an augmentative tool that enhances human judgment, a paradigm described as “high-performance medicine” [3,5].

Within medical education, AI technologies are being adopted to support academic writing, simulation-based learning, assessment development, knowledge synthesis, and clinical reasoning instruction [6]. AI has the potential to reshape medical training through adaptive learning platforms, automated feedback mechanisms, learning analytics, and immersive simulations that enable individualized educational experiences [7,8]. These applications are particularly relevant to competency-based medical education, which relies on continuous assessment and personalized learning pathways to support learner development [9].

A major inflection point occurred with the public release of generative large language models (LLMs), particularly ChatGPT, in late 2022. Unlike prior rule-based or narrow AI systems, LLMs leverage transformer architectures and massive training corpora to generate coherent, context-aware, and conversational responses [9,10]. Early evaluations demonstrated that ChatGPT could perform at a level comparable to medical students or residents on standardized medical examinations, including the United States Medical Licensing Examination (USMLE) [10].

In response, educators have increasingly explored AI integration across undergraduate and graduate medical education for tasks such as content summarization, practice question generation, case simulation, and academic writing assistance [7,8]. Emerging evidence suggests that AI-assisted tools may enhance learner efficiency, engagement, and cognitive load management, particularly in preclinical stages of training [7]. However, reported outcomes remain heterogeneous, reflecting substantial variation in study designs, learner groups, AI tools, and outcome measures across published research. In addition, optimal pedagogical strategies for effective AI integration are still evolving [6].

Despite growing enthusiasm, substantial concerns persist regarding the responsible use of AI in medical education. Ethical issues include academic misconduct, erosion of foundational clinical reasoning skills, limited transparency in AI decision-making processes, algorithmic bias, and risks to data privacy [11,12]. Studies assessing AI literacy among medical students and faculty have demonstrated wide variability in knowledge, confidence, and readiness for adoption [13,14]. Overreliance on AI-generated outputs without appropriate verification may undermine the development of independent diagnostic reasoning, a concern shared by educators and policymakers alike [11,15]. Multiple cross-sectional surveys indicate that students often report higher enthusiasm and earlier adoption of AI tools, whereas faculty tend to express greater ethical concern and stronger calls for governance frameworks [14-16]. These findings underscore the need for structured, evidence-based AI education rather than informal or unregulated implementation [16,17]. Notably, the rapid diffusion of AI tools has frequently outpaced the development of institutional policies, faculty training, and curricular oversight mechanisms [17,18]. Moreover, most published research focuses on allopathic or international programs, leaving osteopathic medical education underrepresented in empirical studies [14-16].

This evidence gap is particularly relevant because osteopathic programs emphasize holistic, patient‑centered training and may face unique considerations regarding the responsible integration of AI into preclinical learning. Without baseline institutional data, it is difficult for osteopathic medical schools to determine appropriate levels of AI literacy training, faculty development, or governance structures to support responsible use.

To address this gap, the present study examined AI knowledge, utilization patterns, perceptions, and future expectations among preclinical osteopathic medical students and faculty at a single institution. The primary objective was to characterize baseline AI literacy, usage behaviors, and attitudes within this population, and the secondary objective was to compare these domains between students and faculty to identify potential group‑level differences in exposure, readiness, and educational needs. These findings provide preliminary, context‑specific insights intended to guide local curricular planning, faculty development, and institutional policy formation related to AI in medical education.

## Materials and methods

Study design and setting

A cross-sectional survey study was conducted at Burrell College of Osteopathic Medicine (BCOM), encompassing both the Las Cruces, New Mexico, and Melbourne, Florida campuses. By including two geographically distinct campuses operating under a unified curricular framework, the study aimed to capture a comprehensive representation of AI engagement across institutional contexts while minimizing curricular heterogeneity. The study followed the Strengthening the Reporting of Observational Studies in Epidemiology (STROBE) guidelines for cross-sectional research to ensure methodological transparency and completeness.

This investigation was designed as an exploratory, descriptive study rather than a hypothesis‑driven analysis. The primary objective of the study was to characterize and compare AI-related knowledge, educational use, attitudes, and future expectations among two key stakeholder groups in preclinical medical education: osteopathic medical students and faculty actively involved in teaching or curricular delivery. The cross-sectional design was selected to provide a timely snapshot of AI-related practices during a period of rapidly expanding access to generative and educational AI tools.

Participants

Eligible participants included first- and second-year osteopathic medical students enrolled in the Class of 2027 and Class of 2028, as well as faculty members engaged in preclinical medical education. Faculty eligibility encompassed both basic science and clinical faculty who contribute to preclinical instruction through lectures, small-group facilitation, assessment development, or curricular design. Inclusion of both instructional and content-expert faculty was intended to reflect the breadth of educational roles influenced by AI technologies.

Inclusion criteria required participants to be aged 21 years or older and currently affiliated with BCOM in one of the eligible roles. Individuals not involved in preclinical education, those below 21 years of age, or those who declined participation were excluded. Participation was entirely voluntary, and no financial or academic incentives were offered.

To promote candid responses and minimize social desirability bias, the survey was administered anonymously. Informed consent was implied through voluntary completion of the survey, consistent with institutional review board (IRB) guidance for exempt educational research.

Sample size estimation

Sample size estimation was conducted a priori using Cochran’s formula for finite populations. Assuming a population size of approximately 545 eligible participants (combined students and faculty), a 95% confidence level (α = 0.05), a margin of error of 5%, and a conservative proportion estimate of 50%, the minimum required sample size was calculated to be 225. Accounting for an anticipated non‑response rate of approximately 10%, a target sample size of 248 participants was established.

However, only 58 complete responses were ultimately available for analysis. As a result, the study did not reach the a priori target and is underpowered for detecting small effect sizes. Given this limitation, the study is framed as an exploratory descriptive analysis, and all inferential findings, particularly nonsignificant comparisons, should be interpreted cautiously. The reduced sample size limits generalizability and statistical power but still provides valuable preliminary institutional insights into AI knowledge, utilization, and perceptions within this osteopathic medical education context.

Survey instrument

Data were collected using a structured, self-administered electronic questionnaire (see Appendices). The survey instrument was adapted, with permission, from a previously validated questionnaire developed by Civaner et al. [14] and used in studies assessing AI needs and perceptions in medical education. The adapted instrument was reviewed for clarity, relevance to the osteopathic medical education context, and alignment with current AI applications.

The finalized survey consisted of 22 items organized into five sections, reflecting the structure of the questionnaire presented in the Appendices:

Demographics

Demographic characteristics included age (years and months), gender (male, female, non‑binary, prefer not to say), respondent role (faculty or student), faculty discipline (basic science or clinical science), and student class year (2027 or 2028).

Knowledge of the Use of AI in Medical Education

This included self‑reported knowledge of AI in medical education; prior formal AI education or training and its modality (elective coursework, conferences/seminars, required coursework, online training, or other); frequency of encountering topics that could be better learned or taught with AI; and use of AI for specific educational, assessment‑related, scholarly, and clinical documentation tasks (e.g., studying or lecture preparation, generating or answering practice questions, creating or reviewing exam items, writing assignments, drafting research papers, case reports, or patient notes). This section also assessed use of AI‑driven educational tools, including digital anatomy, virtual dissection, virtual physiology simulations, computational pathology, high‑fidelity simulations, AI‑generated cases or simulations, and virtual patients.

Perception of the Use of AI in Medical Education

Perceptions of the use of AI in medical education included the perceived overall benefit of AI; specific perceived benefits (e.g., improved diagnostic accuracy, enhanced learning experiences, personalized education plans, administrative efficiency); and concerns regarding AI use (e.g., privacy, ethics, job displacement, over‑reliance on technology).

Attitudes Toward the Use of AI in Medical Education

Attitudes included the preferences for AI compared with traditional information sources; enthusiasm for newer AI tools; perceived impact of AI on medical knowledge, preparation time, and accuracy of practice questions; verification practices; and attitudes toward the need for institutional AI policy.

Future Perspectives

Future perspectives included beliefs about AI’s future role in medical education; areas expected to benefit most from AI integration (diagnostics, treatment planning, patient management); likelihood of recommending AI use; interest in participating in future AI‑related studies or training; and space for additional comments or suggestions.

Survey items included five-point Likert-scale questions, binary and multiple-response selections, and optional open-ended responses. The survey was administered online using Qualtrics (Qualtrics, Provo, UT, USA).

Instrument validation and pilot testing

The survey instrument was adapted from a previously published and validated questionnaire designed to assess knowledge, attitudes, and perceived needs related to AI in allopathic medical education [14]. The original instrument demonstrated acceptable content validity and internal consistency in prior applications among medical learners and educators. Permission to adapt the instrument was obtained, and modifications were made to ensure relevance to the osteopathic medical education context and to reflect contemporary AI applications.

Content validity was addressed through iterative review by the study investigators, which included medical educators with experience in preclinical curriculum development and familiarity with AI-enhanced educational tools. During this process, survey items were evaluated for clarity, relevance, and alignment with the study objectives. Minor wording adjustments were made to improve readability and contextual applicability, while preserving the original conceptual domains of the instrument.

Face validity was further assessed through informal pilot testing with a small convenience sample of preclinical faculty members and students who were not included in the final study cohort. Pilot participants reviewed the survey for comprehensibility, item interpretation, and overall flow, and provided feedback regarding item clarity and response options. Based on this feedback, minor refinements were made to item wording and response formatting to reduce ambiguity and improve user experience.

Given the exploratory nature of the study and the adaptation of an existing validated instrument, formal psychometric revalidation (e.g., factor analysis or reliability testing) was not performed. However, the survey structure and content were deemed appropriate for descriptive and comparative analyses of AI knowledge, utilization, and perceptions among preclinical osteopathic medical students and faculty.

Data collection

Data were collected anonymously between May and July 2025. The first page of the survey included an informed consent statement detailing the study purpose, voluntary nature of participation, risks, benefits, confidentiality protections, and investigator contact information. Consent was implied through voluntary survey completion. No personally identifiable information was collected. The anticipated survey completion time was approximately 15-20 minutes.

Ethical considerations

The study protocol was reviewed and approved by the BCOM Institutional Review Board (IRB No. 0155_2025). The study was granted Exempt Status under Category II (research involving anonymous educational surveys) in accordance with 45 CFR 46.101. The study posed no more than minimal risk to participants. There were no financial costs, incentives, or direct personal benefits associated with participation. All procedures complied with institutional and federal regulations governing human subjects research.

Data handling and management

Survey responses were exported from Qualtrics into Microsoft Excel (Microsoft Corp., Redmond, WA, USA) for preliminary data cleaning and coding, then imported into Python (Python Software Foundation, Wilmington, Delaware, USA) for statistical analysis. Data were stored on secure, password-protected institutional servers accessible only to the study team.

Incomplete responses were included in the calculation of response rates but excluded from inferential statistical analyses. No data imputation was performed, as missing data primarily resulted from early voluntary survey termination rather than sporadic item nonresponse.

Statistical analysis

Data Summary and Descriptive Statistics

Descriptive statistics were used to summarize participant demographics and survey responses. Categorical variables were summarized using frequencies and percentages. Continuous variables, such as age, were summarized using means and standard deviations. All descriptive analyses were stratified by respondent group (faculty vs. students).

Inferential Statistical Analysis

Group comparisons between faculty and students were conducted using chi-square (χ^2^) tests of independence for categorical variables, including AI knowledge levels, training exposure, utilization patterns, perceptions, attitudes, and future perspectives. When expected cell counts were less than five, response categories were collapsed when methodologically appropriate, and results were interpreted cautiously. Age was compared descriptively only, given the non-overlapping nature of student and faculty cohorts.

Because the study was exploratory and descriptive, no adjustments were made for multiple comparisons. We acknowledge that conducting multiple chi-square tests increases the risk of type I error (false-positive findings). Accordingly, statistically significant results are interpreted conservatively, with emphasis placed on effect size, consistency with prior literature, and educational relevance rather than on p‑values alone.

All statistical tests were two-tailed. A p-value < 0.05 was considered statistically significant, and trend-level findings (p < 0.10) were reported when deemed educationally meaningful within the exploratory framework of the study.

Software

All statistical analyses and visualizations were conducted using Python (version 3.x) with standard scientific computing libraries, including pandas, SciPy, and Matplotlib.

Reporting Standards

The study adhered to accepted reporting guidelines for cross-sectional educational research. Given the exploratory design, no adjustments were made for multiple comparisons; findings were interpreted conservatively with emphasis on effect direction, consistency, and educational relevance rather than statistical significance alone.

## Results

Response rate and participant characteristics

Of the 362 surveys distributed, 94 responses were received (26.0%), and 58 complete surveys were included in analyses. Students comprised the majority of respondents. As expected in a preclinical setting, faculty were significantly older than students, and gender distributions differed between groups. These demographic differences provide context for interpreting subsequent comparisons but were not central analytic variables. Detailed demographic characteristics are presented in Table 1.

**Table 1:** Demographic characteristics by analytic group (n = 58) Data are presented as mean ± standard deviation for continuous variables and as frequencies with percentages for categorical variables. Demographic characteristics are summarized descriptively to provide context for faculty and student respondent groups.

Characteristic	Faculty and Students	Faculty	Students
Mean age ± SD (years)	37.2 ± 14.7	54.4 ± 10.0	28.1 ± 5.2
Female, n (%)	32 (55.2)	5 (25.0)	27 (71.1)
Male, n (%)	23 (39.7)	15 (75.0)	8 (21.1)
Non‑binary, n (%)	3 (5.2)	0 (0)	3 (7.9)

Knowledge of the use of AI in medical education

Self‑Reported Knowledge of Use of AI in Medical Education

Respondents across both groups generally described themselves as having only partial knowledge of AI in medical education, with very few indicating high levels of expertise. Faculty and students demonstrated similar distributions of self‑reported knowledge, indicating that baseline AI literacy is modest and comparable across roles (Table 2). This pattern suggests that while awareness of AI is widespread, advanced competency is uncommon, underscoring a shared institutional need for structured, curriculum‑based AI education to support responsible integration into preclinical training.

**Table 2:** Self-reported knowledge of use of AI in medical education (n = 58) Data are presented as n (%). A chi‑square (χ^2^) test of independence evaluated the overall distribution of self‑reported AI knowledge levels between faculty and students. The χ^2^ statistic and p-value reflect comparison across all five knowledge categories collectively.

Knowledge Level	Faculty and Students, n (%)	Faculty, n (%)	Students, n (%)	χ^2^ (df = 4)	p-value
Very knowledgeable	1 (1.7)	0 (0)	1 (2.6)	0.87	0.929
Quite knowledgeable	14 (24.1)	5 (25.0)	9 (23.7)		
Partial knowledge	34 (58.6)	12 (60.0)	22 (57.9)		
Heard of AI only	7 (12.1)	2 (10.0)	5 (13.2)		
No knowledge	2 (3.4)	1 (5.0)	1 (2.6)		

Formal Education or Training in AI

Formal AI training was uncommon across both groups, indicating that most engagement with AI occurs informally rather than through structured institutional offerings. Faculty were significantly more likely than students to report prior AI training, typically through conferences or seminars, yet overall exposure remained low (Table 3). This pattern suggests that despite faculty having somewhat greater formal exposure, both groups lack systematic training, highlighting an institutional opportunity to develop coordinated AI education and professional development initiatives.

**Table 3:** Formal education or training in AI by respondent group (n = 58) Data are presented as n (%). Training modalities are summarized descriptively. *The chi‑square statistic and p‑value are reported only for the collapsed binary comparison (any formal AI training vs. none) due to sparse cell counts in individual training categories.

Training Modality	Faculty and Students, n (%)	Faculty, n (%)	Students, n (%)	χ^2^ (df = 1)*	p-value
Attended online training or courses	1 (1.72)	0 (0.00)	1 (2.63)	-	-
Attended conferences, seminars, or presentations	7 (12.07)	7 (35.00)	0 (0.00)	-	-
Any formal AI training (collapsed)	8 (13.8)	7 (35.0)	1 (2.6)	8.98	0.003
Not received any formal education or training	50 (86.21)	13 (65.00)	37 (97.37)	-	-

Encountering Topics That Can Be Better Learned or Taught With AI

Both faculty and students reported regularly encountering topics they believed could be better supported through AI‑enhanced learning, and their response patterns were similar. This shared perception suggests that opportunities for AI‑supported instruction are already present within the preclinical curriculum. The absence of group differences indicates that interest in AI‑augmented learning is not role‑specific but reflects a broader institutional recognition of areas where AI could enhance teaching and learning (Table 4).

**Table 4:** Encountering topics that could be better learned or taught with AI (n = 58) Data are presented as n (%). A chi‑square (χ^2^) test of independence evaluated the overall distribution of Likert‑scale responses between faculty and students. The χ^2^ statistic and p-value reflect comparison across all response categories collectively.

Response Category	Faculty and Students, n (%)	Faculty, n (%)	Students, n (%)	χ^2^ (df = 4)	p-value
Always	9 (15.52)	3 (15.00)	5 (13.16)	1.04	0.903
Frequently	18 (31.03)	5 (25.00)	12 (31.58)		
Occasionally	17 (29.31)	7 (35.00)	10 (26.32)		
Rarely	10 (17.24)	4 (20.00)	7 (18.42)		
Never	4 (6.90)	1 (5.00)	4 (10.53)		

Ever Used AI in Medical education

Students and faculty demonstrated distinctly different patterns of AI use across academic activities (Table 5). Students reported substantially higher engagement with AI for core learning tasks, including studying, generating and reviewing exam questions, completing written assignments, and drafting patient notes, indicating that AI has become embedded in their routine academic workflow. The most pronounced gap was in documentation‑related activities, where student use was nearly universal.

**Table 5:** Use of AI in medical education by activity (n = 58) Data are presented as n (%). Chi‑square (χ^2^) tests compare faculty and student respondents for each activity independently to evaluate differences in AI utilization patterns.

Application	Faculty, n (%)	Students, n (%)	χ^2^ (df = 1)	p-value
Assist with studying/lecture preparation	9 (45.0)	29 (76.3)	4.39	0.036
Generate practice questions	13 (65.0)	29 (76.3)	0.37	0.544
Answer practice questions	7 (35.0)	23 (60.5)	2.47	0.116
Generate exam questions	4 (20.0)	24 (63.2)	9.41	0.002
Review exam questions	2 (10.0)	27 (71.1)	19.23	<0.001
Generate case scenarios/vignettes	13 (65.0)	19 (50.0)	0.51	0.477
Suggest research topics/questions	6 (30.0)	12 (31.6)	0.00	1.000
Help complete written assignments	0 (0.0)	14 (36.8)	7.81	0.005
Help write research papers	1 (5.0)	6 (15.8)	0.60	0.438
Help write case reports	0 (0.0)	3 (7.9)	0.47	0.492
Help write patient notes	1 (5.0)	35 (92.1)	46.68	<0.001

Faculty, in contrast, used AI more selectively and primarily for instructional purposes such as generating case scenarios or supporting formative assessment development. Both groups reported limited use of AI for scholarly writing or research activities. Overall, these patterns suggest that students are adopting AI as a broad learning and productivity tool, whereas faculty engagement remains narrower and more pedagogically focused, highlighting a growing divergence in how each group incorporates AI into educational practice.

Ever Used AI‑driven Educational Tools in Medical Education

Use of AI‑enhanced educational tools varied across platforms (Table 6), with digital anatomy resources showing the highest adoption, particularly among students. In contrast, other tools such as virtual dissection platforms, physiology simulations, and more advanced AI‑enabled applications (e.g., computational pathology or high‑fidelity simulations) were used infrequently by both groups. These patterns suggest that AI integration within the preclinical curriculum is currently concentrated in supplemental learning tools rather than in more sophisticated simulation or clinical reasoning technologies, reflecting early‑stage adoption and limited institutional exposure to advanced AI‑driven platforms.

**Table 6:** Use of AI driven educational tools (n = 58) Data are presented as n (%). Chi‑square (χ^2^) tests compare faculty and student respondents for each educational tool independently to assess differences in adoption patterns.

Educational Tool	Faculty, n (%)	Students, n (%)	χ^2^ (df = 1)	p-value
Digital anatomy platforms	7 (35.0)	31 (81.6)	10.61	0.001
Virtual dissection tools	5 (25.0)	18 (47.4)	1.88	0.170
Virtual physiology simulations	4 (20.0)	8 (21.1)	0.00	1.000
Computational pathology tools	0 (0.0)	3 (7.9)	0.44	0.505
High‑fidelity simulations	1 (5.0)	3 (7.9)	0.00	1.000
AI‑generated cases or simulations	2 (10.0)	4 (10.5)	0.00	1.000

Perception of the use of AI in medical education

Perceived Benefit of the Use of AI in Medical Education

Faculty and students expressed similarly positive views regarding the educational value of AI (Table 7), with most respondents rating it as highly beneficial. The absence of group differences indicates broad, shared support for AI’s role in enhancing learning. This alignment suggests that enthusiasm for AI integration is not limited to a particular stakeholder group but reflects a general institutional readiness to incorporate AI‑enabled tools into preclinical education.

**Table 7:** Perceived benefit use of AI in medical education (n = 58) Data are presented as n (%). A chi‑square (χ^2^) test of independence evaluated the overall distribution of perceived benefit ratings across all response categories.

Response Category	Faculty and Students, n (%)	Faculty, n (%)	Students, n (%)	χ^2^ (df = 4)	p-value
Extremely	15 (25.86)	4 (20.00)	11 (28.95)	4.72	0.318
Very	23 (39.66)	7 (35.00)	16 (42.11)		
Moderately	17 (29.31)	9 (45.00)	8 (21.05)		
Slightly	2 (3.45)	0 (0.00)	2 (5.26)		
Not at all	1 (1.72)	0 (0.00)	1 (2.63)		

Potential Benefits of Integrating AI Into Medical Education

Respondents across both groups identified similar advantages of AI integration, most commonly highlighting enhanced learning experiences, personalized education, and administrative efficiency (Table 8). Faculty were somewhat more likely to view AI as improving diagnostic accuracy, though this difference was not statistically significant. Overall, the pattern of responses indicates broad agreement between faculty and students regarding the educational value of AI, suggesting shared expectations about how AI could meaningfully support teaching and learning within the preclinical curriculum.

**Table 8:** Potential benefits of AI integration into medical education (n = 58; multiple responses allowed) Data are presented as n (%). Chi‑square (χ^2^) tests compare faculty and student respondents for each perceived benefit independently to assess differences in endorsement patterns.

Benefit	Faculty, n (%)	Students, n (%)	χ^2^ (df = 1)	p-value
Enhanced learning experiences	15 (75.0)	29 (76.3)	0.00	1.000
Administrative efficiency	10 (50.0)	16 (42.1)	0.09	0.767
Personalized education plans	8 (40.0)	18 (47.4)	0.07	0.796
Improved diagnostic accuracy	9 (45.0)	9 (23.7)	1.87	0.171

Concerns About the Use of AI in Medical Education

Respondents expressed a wide range of concerns about AI in medical education, with overreliance on technology, ethical issues, and privacy emerging as the most prominent themes. Although faculty tended to report stronger concern about ethical risks and overreliance than students, these differences did not reach statistical significance. Students expressed comparable levels of concern regarding privacy and workforce implications.

Overall, the pattern suggests that while concerns about AI are broadly shared across roles, faculty place relatively greater emphasis on ethical safeguards and responsible use, whereas students’ concerns are more evenly distributed (Table 9). These findings highlight the importance of developing institutional policies that address both ethical oversight and practical implementation challenges as AI becomes more integrated into preclinical education.

**Table 9:** Concerns about the use of AI in medical education (n = 58; multiple responses allowed) Data are presented as n (%). Each concern was analyzed independently as a binary variable (selected vs. not selected) using chi‑square (χ^2^) tests of independence to compare faculty and student respondents. Categories with sparse cell counts (≤3%) were summarized descriptively and not subjected to inferential testing.

Concern	Faculty and Students, n (%)	Faculty, n (%)	Students, n (%)	χ^2^ (df = 1)	p-value
Privacy issues	28 (48.28)	9 (45.00)	19 (50.00)	0.01	0.932
Ethical concerns	31 (53.45)	15 (75.00)	17 (44.74)	3.71	0.054
Over‑reliance on technology	40 (68.97)	18 (90.00)	25 (65.79)	2.84	0.092
Job displacement	17 (29.31)	4 (20.00)	13 (34.21)	0.68	0.408
Other	1–2 (≤3%)	≤1 (≤5%)	≤1 (≤3%)	-	Descriptive

Areas of Medical Education That Would Benefit Most From AI Integration

Faculty and students identified different domains of medical education where AI could have the greatest impact (Table 10). Faculty most often pointed to diagnostic reasoning, whereas students more frequently highlighted treatment planning, a difference that reached statistical significance. Perceptions of AI’s value for patient management were similar across groups.

**Table 10:** Areas of medical education perceived to benefit from AI (n = 58) Data are presented as n (%). Each domain was analyzed independently using chi‑square (χ^2^) tests of independence to compare faculty and student respondents.

Area	Faculty and Students, n (%)	Faculty, n (%)	Students, n (%)	χ^2^ (df = 1)	p-value
Diagnostics	30 (51.72)	14 (70.00)	16 (42.11)	3.04	0.081
Patient management	15 (25.86)	5 (25.00)	10 (26.32)	0.00	1.000
Treatment planning	13 (22.41)	1 (5.00)	12 (31.58)	3.90	0.048

These patterns suggest role‑based distinctions in how AI is viewed: faculty emphasize AI’s potential to support diagnostic decision‑making, while students focus more on its relevance to therapeutic planning and future clinical responsibilities. Despite these differences, both groups expressed broad optimism about AI’s contributions to medical education.

Attitudes toward the use of AI in medical education

Faculty and students expressed broadly similar attitudes toward AI, with strong agreement across both groups that AI‑generated output requires verification and that institutional policies are needed to guide responsible use (Table 11). Although faculty showed somewhat stronger support for formal policy development, this difference was not statistically significant. Students were more inclined to view AI as a helpful supplement to traditional information sources and as a tool that may enhance the accuracy of practice questions, but these trends also did not reach significance.

**Table 11:** Attitudes toward use of AI in medical education (agreement vs. non-agreement) (n = 58) Agreement was defined as strongly agree or agree. Chi‑square (χ^2^) tests compared agreement versus non‑agreement (neutral/disagree/strongly disagree) between faculty and students for each attitude statement.

Attitude Statement	Faculty Agree (%)	Students Agree (%)	χ^2^ (df = 1)	p-value
Prefer AI over Google/medical references	20.0	44.7	2.48	0.115
Look forward to newer AI tools	50.0	31.6	1.19	0.276
AI improves my medical knowledge	50.0	63.2	0.47	0.492
AI reduces lecture/exam preparation time	45.0	52.6	0.08	0.782
AI improves the accuracy of practice questions	40.0	65.8	2.58	0.108
AI answers must be verified	95.0	89.5	0.05	0.825
Medical schools should develop AI policies	95.0	55.6	3.24	0.072

Overall, the findings indicate substantial alignment in attitudes toward AI, with faculty placing slightly greater emphasis on governance and oversight, while students focus more on AI’s practical utility. This shared perspective suggests a favorable environment for developing institution‑wide guidelines and educational strategies for AI integration.

Future perspectives on the use of AI in medical education

Faculty and students expressed similarly strong optimism about AI’s future role in medical education (Table 12). Most respondents anticipated that AI would become increasingly important and indicated willingness to recommend its use, with high interest in participating in future AI‑related training or research. The absence of significant group differences suggests a shared readiness for continued AI integration.

**Table 12:** Future perspectives on use of AI in medical education (n = 58) Agreement was defined as strongly agree/agree or very likely/likely, as appropriate. Chi‑square (χ^2^) tests compared faculty and student respondents for each future‑oriented item independently.

Future Perspective Item	Faculty Agree (%)	Students Agree (%)	χ^2^ (df = 1)	p-value
Believe AI will play a significant future role	95.0	81.6	1.02	0.313
Likely to recommend AI use in medical education	60.0	73.7	0.60	0.440
Interested in future AI‑related studies or training	90.0	78.9	0.48	0.488

Although faculty showed slightly stronger endorsement of AI’s future role and students expressed marginally greater willingness to recommend AI, these trends were not statistically significant. Overall, the findings reflect broad, cross‑group enthusiasm for AI’s expanding role in medical education and support the development of future institutional initiatives to guide its implementation.

Qualitative Future‑Oriented Comments

A small number of respondents provided open‑ended comments regarding future AI use. These comments emphasized AI as a form of augmented intelligence, highlighted ongoing concerns about accuracy, reliability, privacy, and ethical safeguards, and reiterated the need for formal institutional policies before broader adoption. Although limited in number, these qualitative insights reinforce the quantitative findings of strong enthusiasm for AI’s future role tempered by a desire for responsible governance.

Overall, these results indicate widespread optimism and readiness for future AI integration among both faculty and students, with no statistically significant differences between groups.

## Discussion

This study provides one of the first systematic examinations of AI knowledge, utilization patterns, and perceptions among preclinical osteopathic medical students and faculty, addressing a notable gap in the medical education literature. Overall, respondents demonstrated moderate familiarity with AI technologies and expressed strong optimism regarding future integration into medical education. These findings are consistent with prior studies conducted in allopathic and international training settings, suggesting that interest in AI adoption extends across institutional structures and geographic contexts [15,17]. Collectively, this convergence reflects broader cultural and technological shifts shaping contemporary health professions education.

Patterns of AI knowledge and training

Despite widespread exposure to AI tools, particularly generative LLMs such as ChatGPT, formal or structured training in AI was uncommon among both students and faculty. These findings align with prior studies demonstrating that AI competence among medical trainees is frequently self-directed rather than institutionally supported [15-17]. Importantly, the observed discrepancy between high utilization and low formal training represents a critical educational vulnerability, as learners may engage with AI tools without sufficient understanding of their limitations, risks, and appropriate applications. Notably, no significant difference was observed in self-reported AI knowledge between faculty and students, despite faculty reporting slightly greater exposure to formal training opportunities. Similar discrepancies have been reported in other educational contexts, suggesting that limited or episodic training does not consistently translate into perceived expertise, particularly when instruction lacks longitudinal integration or applied pedagogical focus [11].

The persistence of partial AI knowledge across both groups highlights an important educational gap. As AI tools increasingly influence learning, assessment, and clinical reasoning, reliance on informal or unstructured engagement may contribute to uneven skill development and widening knowledge disparities [6,19]. In response, several authors have advocated for structured AI literacy curricula that address foundational principles, validation of AI-generated outputs, bias recognition, and ethical use as core components of undergraduate medical education [19,20].

Differential AI utilization between students and faculty

A key finding of this study was the significantly higher utilization of AI by students compared with faculty, particularly for studying and lecture preparation. This pattern mirrors existing literature demonstrating that students are often early adopters of educational technologies, motivated by efficiency, accessibility, and familiarity with digital tools [15,21]. Generational digital fluency and the prominence of self-directed learning in preclinical education may further contribute to this trend, particularly in environments characterized by large volumes of content and high-stakes assessments [21,22].

In contrast, faculty respondents reported lower levels of direct AI use but expressed greater concern regarding ethical implications and the risk of overreliance. This divergence likely reflects differing professional roles: students often prioritize efficiency and exam preparation, whereas faculty are responsible for maintaining educational standards, assessment validity, and professional integrity [14,23].

Perceived benefits and educational value of AI

Across both groups, AI was widely perceived as beneficial, particularly for enhancing learning experiences and supporting personalized education. These findings reinforce growing evidence that AI-enabled tools can facilitate adaptive learning, timely feedback, and individualized study strategies, especially in preclinical curricula [8,24,25]. High endorsement of administrative efficiency further suggests that respondents recognize AI’s potential to reduce cognitive and clerical burden, consistent with broader healthcare trends emphasizing workflow optimization and efficiency [2,26,27].

At the same time, respondents consistently emphasized the importance of verifying AI-generated information, reflecting appropriate caution and awareness of current AI limitations. This balance of optimism and skepticism aligns with the emerging consensus that AI should function as a supportive cognitive tool rather than an authoritative source, particularly in complex medical reasoning and decision-making tasks [21,26,27].

Ethical concerns and governance implications

Concerns related to overreliance on technology, ethical integrity, and data privacy were prevalent among respondents, echoing ethical frameworks proposed in the broader AI-in-healthcare literature [14,16,17,23]. Uncritical dependence on AI-generated content may risk undermining the development of foundational skills such as critical thinking, clinical reasoning, and professional judgment if appropriate safeguards are not in place [19,28]. Ethical considerations, including algorithmic opacity, bias, and data security, further underscore the need for transparent governance structures and institutional oversight [12,26,27].

Given the exploratory design, modest sample size, and single-institution context, the implications of these findings should be interpreted cautiously. The patterns observed reflect local experiences of preclinical students and faculty rather than generalizable trends across medical education. Accordingly, the governance and curricular recommendations that follow are intended to guide institutional reflection within similar contexts rather than serve as prescriptive models for broader adoption.

Faculty respondents demonstrated stronger support for the development of formal institutional AI policies, consistent with their heightened ethical concerns and leadership responsibilities. Prior studies similarly report that educators are more likely than students to advocate for clear guidelines governing AI use in academic environments [14,17]. Well-defined institutional policies can help clarify acceptable use, promote academic integrity, and ensure alignment with accreditation standards and professional values [12,26-28].

Implications for osteopathic medical education

Given the limited representation of osteopathic medical education in AI-related research, these findings carry particular importance. Osteopathic training emphasizes holistic, patient-centered care, integrative clinical reasoning, and humanistic values, which may influence both perceptions of and expectations for AI-assisted learning [29,30]. Understanding baseline AI knowledge and attitudes among osteopathic students and faculty is therefore essential for designing educational strategies that integrate technological innovation while preserving core professional principles. Intentional, values-aligned integration of AI may help ensure that emerging technologies enhance, rather than diminish, the humanistic and reasoning-based competencies central to osteopathic medical education.

Summary and future directions

This study demonstrates that preclinical osteopathic medical students and faculty exhibit strong enthusiasm for AI in medical education, coupled with moderate self-reported knowledge and widespread informal use of AI tools. While respondents across both groups recognized substantial educational benefits, particularly in enhanced learning efficiency, personalization, and academic support, formal training in AI and institutional governance structures were notably limited. This mismatch between rapid adoption and limited preparedness highlights a critical gap between enthusiasm for AI and readiness for its responsible, ethical, and pedagogically sound integration.

The findings underscore the need for comprehensive, structured AI education embedded within undergraduate osteopathic medical curricula. Such initiatives should extend beyond basic tool familiarity and emphasize foundational AI concepts, validation of AI-generated outputs, bias recognition, ethical considerations, and appropriate integration into clinical reasoning and assessment practices. Parallel faculty development programs are essential to equip educators with the knowledge and confidence required to guide learners, design AI-informed assessments, and establish consistent expectations for AI use.

Future research should move beyond cross-sectional assessments to longitudinal studies evaluating the educational and professional impacts of AI-integrated curricula, including effects on clinical reasoning development, diagnostic accuracy, and learner autonomy. Multicenter studies involving multiple osteopathic medical schools are needed to enhance generalizability and capture institutional variability. Additionally, mixed-methods approaches incorporating qualitative data may further elucidate contextual factors influencing AI adoption, resistance, and ethical concern.

Limitations

Several limitations should be considered when interpreting these findings. First, this study was conducted at a single osteopathic medical institution, which limits generalizability to programs with different curricular structures, faculty development priorities, or access to AI resources. Second, the modest sample size reduces statistical power and may limit the ability to detect smaller between‑group differences. Third, the response rate introduces the possibility of nonresponse bias, as individuals with stronger opinions or greater interest in AI may have been more likely to participate.

Fourth, all data were derived from self‑reported survey responses, which are inherently susceptible to recall bias, response bias, and social desirability effects. Fifth, the cross‑sectional design precludes inference about causal or temporal relationships between AI knowledge, utilization, perceptions, and attitudes. Finally, given the rapid evolution of AI technologies and institutional policies, the durability of these findings may be limited, underscoring the need for ongoing reassessment as educational environments continue to change.

## Conclusions

Preclinical osteopathic medical students and faculty demonstrated moderate AI knowledge alongside strong perceived educational benefits and broad openness to future integration. Students reported substantially higher use of AI tools, whereas faculty expressed relatively greater ethical concern and emphasis on oversight. These preliminary, institution‑specific patterns highlight the need for structured AI literacy education, faculty development, and clear governance mechanisms to support responsible and pedagogically sound integration.

Within this context, developing formal AI curricula, investing in faculty training, and establishing institution‑level policies will be important steps to ensure that AI is incorporated ethically, responsibly, and in alignment with the values of osteopathic medical education. Such efforts can help AI function as an augmentative educational tool, enhancing learning while preserving core competencies in clinical reasoning, professionalism, and patient‑centered care.

## Appendices

**Table 13:** Survey questionnaire items and response options The survey questionnaire items and response options were adapted, with permission, from a previously validated questionnaire developed by Civaner et al. [14] for assessing AI needs and perceptions in medical education. All modifications were made to align the instrument with preclinical osteopathic medical education.

Section 1: Demographic																					
What is your age?							Years:								Months:						
What is your gender?							Male								Female						
							Non-binary								Prefer not to say						
Are you a faculty or student?							Faculty								Student						
If you are a faculty member, which of the following do you belong to?							Basic science								Clinical science						
If you are a student, which class year of medical school are you in?							2027								2028						
Section 2: Knowledge of use of AI in Medical Education																					
Rate your knowledge of use of AI in medical education																					
Very knowledgeable		Quite knowledgeable				Partial knowledgeable					Heard about it but possess no knowledge								No knowledge		
Have you ever received any formal education or training in AI?																					
Yes								No													
If yes, please specify the type of education or training in AI received:																					
I took it as an elective course.																				Yes	No
I attended events such as conferences, seminars, and presentations.																				Yes	No
It was included as a subject in a required course in college.																				Yes	No
I attended online training or courses.																				Yes	No
Other (specify):																					
How often do you encounter topics in your medical studies or teaching that can be better learned or taught with assistance of AI-related technologies?																					
Always	Frequently				Occasionally					Rarely								Never			
Have you ever used AI for any of the following in your current role?																					
To assist with studying or lecture preparation							Yes					No				I don’t know how to use					
To generate practice questions							Yes					No				I don’t know how to use					
To answer practice questions							Yes					No				I don’t know how to use					
To generate exam questions							Yes					No				I don’t know how to use					
To review exam questions							Yes					No				I don’t know how to use					
To generate case scenarios or vignettes							Yes					No				I don’t know how to use					
To suggest research topics or questions							Yes					No				I don’t know how to use					
To help complete written assignments							Yes					No				I don’t know how to use					
To help write research papers							Yes					No				I don’t know how to use					
To help write case reports							Yes					No				I don’t know how to use					
To help write patient notes							Yes					No				I don’t know how to use					
Any other (specify):																					
5. Have you ever used any of the following in medical school?																					
Digital anatomy							Yes						No			I don’t know how to use					
Virtual dissection							Yes						No			I don’t know how to use					
Virtual physiology simulations							Yes						No			I don’t know how to use					
Computational pathology							Yes						No			I don’t know how to use					
High fidelity simulations							Yes						No			I don’t know how to use					
AI Generated cases of simulations							Yes						No			I don’t know how to use					
Virtual patients							Yes						No			I don’t know how to use					
Any other (specify):																					
Section 3: Perception of use of AI in medical education																					
1. How beneficial do you think AI is in the field of medical education?																					
Extremely		Very		Moderately					Slightly								Not at all				
2. In your opinion, what are the potential benefits of integrating AI into medical education (select all that apply)																					
Improved diagnostic accuracy							Enhanced learning experience														
Personalized education plans							Increased efficiency in administrative tasks														
Other (specify):																					
3. What concerns do you have about the use of AI in medical education (select all that apply)																					
Privacy issues							Ethical concerns														
Job displacement							Over-reliance on technology														
Other (specify):																					
Section 4: Attitudes toward use of AI in medical education																					
1. I prefer using AI tools rather than Google or another search engine or medical reference to explain a medical topic.																					
Strongly agree		Agree				Neutral					Disagree								Strongly disagree		
2. I always look forward to using newer or updated versions of AI tools.																					
Strongly agree		Agree				Neutral					Disagree								Strongly disagree		
3. Use of AI in medical education improves my knowledge in medicine.																					
Strongly agree		Agree				Neutral					Disagree								Strongly disagree		
4. Use of AI in medical education reduces the time for lecture or exam preparation.																					
Strongly agree		Agree				Neutral					Disagree								Strongly disagree		
5. Use of AI enables me to generate or answer practice questions more accurately.																					
Strongly agree		Agree				Neutral					Disagree								Strongly disagree		
6. Answer provided by AI needs to be verified																					
Strongly agree		Agree				Neutral					Disagree								Strongly disagree		
7. Medical schools should develop policies about the use of AI in medical education																					
Strongly agree		Agree				Neutral					Disagree								Strongly disagree		
Section 5: Future Perspectives																					
1. Do you believe AI will play a significant role in the future of medical education?																					
Strongly agree		Agree				Neutral					Disagree								Strongly disagree		
2. What areas of medical education do you think would benefit most from AI integration?																					
Diagnostics			Treatment planning											Patient management							
3. How likely are you to recommend the use of AI in medical education?																					
Very likely		Likely				Neutral					Unlikely								Very unlikely		
4. Would you be interested in participating in further studies or training programs focused on AI in medical education?																					
Strongly agree		Agree				Neutral					Disagree								Strongly disagree		
5. Do you have any additional comments or suggestions regarding the use of AI in medical education?																					
Comments/Suggestions:																					
